# Clinical Efficacy of Various Diagnostic Tests for Small Bowel Tumors and Clinical Features of Tumors Missed by Capsule Endoscopy

**DOI:** 10.1155/2015/623208

**Published:** 2015-07-02

**Authors:** Jung Wan Han, Sung Noh Hong, Hyun Joo Jang, Seong Ran Jeon, Jae Myung Cha, Soo Jung Park, Jung Sik Byeon, Bong Min Ko, Eun Ran Kim, Hwang Choi, Dong Kyung Chang

**Affiliations:** ^1^Division of Gastroenterology, Department of Internal Medicine, Dongtan Sacred Heart Hospital, Hallym University College of Medicine, 40 Sukwoo-dong, Hwaseong-si, Gyeonggi-do 445-170, Republic of Korea; ^2^Department of Gastroenterology, Samsung Medical Center, Sungkyunkwan University School of Medicine, Seoul, Republic of Korea; ^3^Department of Gastroenterology, Seoul Soon Chun Hyang University Hospital, Soon Chun Hyang University College of Medicine, Seoul, Republic of Korea; ^4^Department of Gastroenterology, Kyung Hee University Hospital at Gangdong, Kyung Hee University College of Medicine, Seoul, Republic of Korea; ^5^Department of Gastroenterology, Yonsei University College of Medicine, Seoul, Republic of Korea; ^6^Department of Gastroenterology, Asan Medical Center, University of Ulsan College of Medicine, Seoul, Republic of Korea; ^7^Department of Gastroenterology, Bucheon Soon Chun Hyang University Hospital, Soon Chun Hyang University College of Medicine, Bucheon, Republic of Korea; ^8^Department of Gastroenterology, Incheon St. Mary's Hospital, Catholic University School of Medicine, Incheon, Republic of Korea

## Abstract

*Background*. We aimed to evaluate the efficacy of various diagnostic tools such as computerized tomography (CT), small bowel follow-through (SBFT), and capsule endoscopy (CE) in diagnosing small bowel tumors (SBTs). Additionally, we aimed to evaluate the clinical features of SBTs missed by CE. *Methods*. We retrospectively studied 79 patients with histologically proven SBT. Clinical data were analyzed with particular attention to the efficacy of CT, SBFT, and CE in detecting SBT preoperatively. We also analyzed the clinical features of SBTs missed by CE. *Results*. The most common symptoms of SBT were bleeding (43%) and abdominal pain (13.9%). Diagnostic yields were as follows: CT detected 55.8% of proven SBTs; SBFT, 46.1%; and CE, 83.3%. The sensitivity for detecting SBTs was 40.4% for CT, 43.9% for SBFT, and 79.6% for CE. Two patients with nondiagnostic but suspicious findings on CE and seven patients with negative findings on CE were eventually found to have SBT. These nine patients were eventually diagnosed with gastrointestinal stromal tumor (4), small polyps (3), inflammatory fibroid polyp (1), and adenocarcinoma (1). These tumors were located in the proximal jejunum (5), middle jejunum (1), distal jejunum (1), and proximal ileum (1). *Conclusion*. CE is more efficacious than CT or SBFT for detecting SBTs. However, significant tumors may go undetected with CE, particularly when located in the proximal jejunum.

## 1. Introduction 

The small intestine represents 75% of the length and 90% of the absorptive surface area of the gastrointestinal system. However, small bowel tumors (SBTs) are rare, representing only 3–6% of gastrointestinal (GI) tract tumors and only 1–3% of all malignant GI tumors [1]. The diagnosis of SBTs is difficult and is frequently delayed. Conventional diagnostic modalities are inaccurate and inconclusive [2] and frequently fail to detect early or locally advanced stages because of the inaccessibility of the small bowel. Indirect evaluation is possible with small bowel follow-through (SBFT) or computed tomography (CT), but disadvantages include patient discomfort, high radiation dose, and, most importantly, poor sensitivity in detecting SBTs [3, 4].

Capsule endoscopy (CE) allows painless endoscopic imaging of the entire small bowel. CE has rapidly gained acceptance as a standard method for small bowel evaluation even though it is contraindicated in patients with suspected or documented intestinal obstruction and does not allow therapeutic intervention [5, 6]. In large cohort undergoing CE, the prevalence of SBT ranges from 2 to 9%, higher than previously reported [7–14]. CE may provide the earlier diagnosis and treatment of SBTs compared to comparative methods [8, 15]. However, some studies revealed that CE can miss some significant tumor in the small bowel [16–18].

This study aimed to investigate the characteristics of SBTs confirmed by pathology, to compare diagnostic yields among various diagnostic methods, and to identify the characteristics of SBTs missed by CE.

## 2. Materials and Methods 

The records of 79 patients with histology proven SBTs by surgery (43 patients) or double balloon enteroscopy (DBE, 65 patients) from March 2004 to December 2012 were retrospectively studied. Patients had been treated at 7 medical referral centers in Korea and had undergone surgery or DBE for SBT removal. We reviewed the clinical characteristics of SBTs; the diagnostic yields of SBFT, CT, and CE; and the characteristics of SBTs not detected by CE. Of these 79 patients, 39 (49.3%) underwent SBFT, 68 (86.1%) underwent CT, and 54 (68.3%) underwent CE before SBTs were confirmed. Findings of these diagnostic studies were interpreted by experienced endoscopists and radiologists, and the results were classified into three categories: definite, suspicious, or negative. When SBFT, CT, or CE revealed findings considered confirmatory of a diagnosis of SBT, the results were classified in the “definite SBT” category. When findings were suggestive but not confirmatory of SBT, the results were classified as “suspicious SBT.” When no evidence of neoplasia was found, the results were classified as “negative.” Locations of SBTs were classified as duodenum, jejunum (proximal, mid, or distal), and ileum (proximal, mid, or distal). In patients operated on, the locations of SBTs were classified according to operative findings. In patients nonoperated on, we approximately estimated the locations of SBTs with the depth of insertion of enteroscope, the size of small intestinal lumen, and the shape of villi and folds.

### 2.1. Capsule Endoscopy

The PillCam SB video (SB1 and SB2, Given Imaging, Yokneam, Israel) and MiroCam (IntroMedic, Seoul, South Korea) were used for CE. Polyethylene glycol solution (2–4 L) was administered before the examination for cleansing and enhancement of visual clarity. Video findings were interpreted by experienced gastrointestinal endoscopists at each center.

### 2.2. Double Balloon Enteroscopy

DBE was performed using EN-450P5/20 or EN-450T5 (Fujinon Inc., Saitama, Japan) under conscious sedation. All procedures were carried out by experienced endoscopists. The technique for insertion has been previously described [19]. Enteroscopic exploration was discontinued when the targeted lesion was reached. Total enteroscopy was confirmed by reaching the cecum, or by reaching an India ink stain previously made through the other approach. Biopsy, polypectomy, or endoscopic mucosal resection specimens obtained by DBE were reviewed by experienced pathologists. The protocol was approved by the Institutional Review Board at each institute.

### 2.3. Statistical Analysis

SPSS 11.5 (SPSS Inc., Chicago, IL) was used for all statistics. Continuous variables are presented as means ± standard deviations and were analyzed using ANOVA. The comparisons of clinical factors affecting diagnostic yields of SBTs by CT, SBFT, and CE were assessed by using chi-square test and Fisher exact test. The proportions of patients with positive findings at two examinations were compared, and a significant difference between the tests was calculated by using the exact McNemar test. A *P* value < 0.05 was considered statistically significant.

## 3. Results

### 3.1. Clinicopathologic Characteristics of SBTs

**Figure 1:**
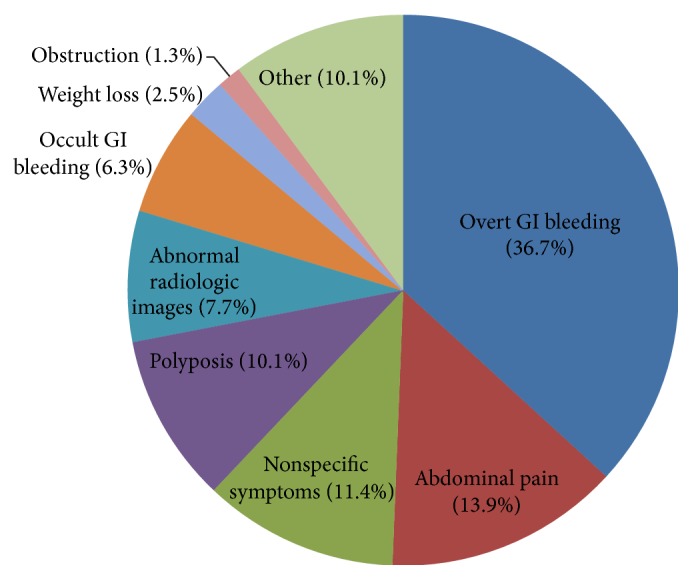
Various indications of diagnostic tests for small bowel tumors.

The clinical characteristics of patients are presented in Table 1. The incidence of SBTs was higher in men (63.3%) than in women (36.7%). Forty-five (57%) of 79 patients had anemia, and the mean hemoglobin level was 10.1 ± 2.9 g/dL. The mean size of SBTs was 3.5 ± 2.6 cm, and 54 patients (68.3%) had a single lesion. SBTs were localized in 68 patients (86.1%) and locally advanced in 6 patients (7.6%) and metastatic disease was present in 5 patients (6.3%). The most frequent symptom was overt GI bleeding (36.7%) and abdominal pain (13.9%) (Figure 1). Of 43 patients who were treated by surgery, 29 patients underwent DBE and surgery and 14 patients underwent surgery without DBE. The size of tumors in the patients with DBE and surgery was significantly smaller than the patients diagnosed by surgery without DBE (3.31 ± 2.33 cm versus 5.88 ± 2.93 cm, *P* = 0.031).

### 3.2. Locations of SBTs according to Diagnosis

The SBTs were benign in 63 patients (79.7%) and malignant in 16 patients (20.3%). Benign tumors were leiomyoma (24/52, 46.2%), hamartoma (17/52, 32.7%), benign polyps (7/52, 13.5%), lipoma (3/52, 5.8%), and hemangioma (1/51, 2.9%). Malignant tumors were adenocarcinoma (7/16, 43.8%), gastrointestinal stromal tumor (GIST) (5/16, 31.3%), lymphoma (3/16, 18.8%), and carcinoid tumor (1/16, 6.3%). Both benign and malignant tumors occurred most frequently in the proximal jejunum (34/79, 43.0%) followed by the distal ileum (9/79, 11.4%), duodenum (8/79, 10.1%), middle jejunum (6/79, 7.6%), distal jejunum (5/79, 6.3%), proximal ileum (4/79, 5.1%), and middle ileum (3/79, 3.8%).

### 3.3. Diagnostic Yields of SBFT, CT, and CE for SBTs

The diagnostic yields for definitive SBTs were 55.8% (38/68) in CT, 46.1% (18/39) in SBFT, and 83.3% (45/54) in CE (Table 2). Using DBE as a reference in the 95% confidence interval (CI), the sensitivity of each diagnostic method was 40.4% (95% CI, 27.01–54.90%) in CT, 43.9% (95% CI, 28.47–60.25%) in SBFT, and 79.6% (95% CI, 65.66–89.76%) in CE. A significant difference was found between diagnostic yields of CE and SBFT (36.36%; 95% CI, 9.36–50.64%; *P* = 0.0075) and between those of CE and CT (33.33%, 95% CI, 12.75–43.82%; *P* = 0.0015) (Table 3). The clinical factors affecting diagnostic yields were the presence of anemia (*P* = 0.019) and bleeding (*P* = 0.043) for CT and tumor size >10 mm (*P* = 0.021) for SBFT (Table 4). There were no statistically significant clinical factors affecting diagnostic yields of CE.

There were 32 patients who had all three modalities (CT, SBFT, and CE). We compared the sensitivities of these three modalities in 32 patients. It showed that the sensitivity of CE for SBTs is significantly higher than SBFT or CT (Table 5).

### 3.4. Characteristics of SBTs Missed by CE

In nine (16.7%) of 54 patients, SBTs were not detected by CE but were eventually diagnosed by DBE. Seven patients had negative findings and two patients had suspicious findings on CE. SBTs >10 mm were identified in six patients (66.7%). Most of the missed SBTs were located in the proximal jejunum (5, 55.6%), followed by the proximal ileum (2, 22.2%), middle jejunum (1, 11.1%), and distal jejunum (1, 11.1%). The final diagnoses in these nine patients were GIST (4, 44.4%), small polyps (3, 33.3%), adenocarcinoma (1, 11.1%), and inflammatory fibroid polyp (1, 11.1%).

## 4. Discussion 

In this study, we demonstrated that the most common SBT was leiomyoma for benign tumor and adenocarcinoma for malignancy, and the most common location of SBTs was proximal jejunum in Korea. The diagnostic yield and sensitivity of CE was higher than SBFT and CT scan. We also found that the SBTs missed by CE were 16.7%, missed SBTs were mostly located in proximal jejunum, and GIST was the most common histologic type of missed SBTs.

Primary small bowel neoplasms are rare accounting for only 3% of all gastrointestinal tract neoplasia in the United States [20]. In Korea, the 2008 annual report of cancer statistics showed that small intestinal cancer accounted for 0.98% of all GI malignancies [21]. However, several studies have reported an increasing incidence of SBTs [22, 23]. SBTs are often diagnosed too late for successful treatment because clinical manifestations of SBTs are usually nonspecific and conventional radiologic tests have some limitations to detect small sized SBTs.

The study from the United States showed that carcinoid tumor is the most common SBTs rather than adenocarcinoma [24]. Cangemi et al. reported that the most common SBT was carcinoid tumor (19.4%) followed by GIST and lymphoma [25]. They also reported that hamartoma (10.4%) was the most common benign tumor, followed by inflammatory polyps and adenoma. In Japan, lymphoma (21.5%) and GIST (18.8%) were the most common tumors, and carcinoid tumors were rare (2.8%) [26]. In recent Korean studies, carcinoid tumor is rare in the small intestine [27, 28]. This difference is presumed to be a result of genetic and ethnic factors. Primary adenocarcinoma is the most common SBT detected by DBE in another Japanese study [29]. In a Korean study of 112 patients with SBTs, the most common malignancy was GIST/leiomyoma and the most common benign polyp was hamartoma [29]. In our study, leiomyoma and GIST were categorized separately, and the most common benign SBT was leiomyoma. Adenocarcinoma was the most common malignancy in our study. Further studies are needed to accurately determine the frequency of SBTs in Korea.

The most common location of SBTs is the ileum in a previous study of the United States [25]. Our study found that the distribution of SBTs is similar to that of a previous Korean study, which reported that SBTs were most frequently located in the jejunum (61.7%), followed by ileum (34.9%) and duodenum (16.1%) [29].

Diagnostic radiology studies such as SBFT, enteroclysis, and CT were traditionally used to diagnose SBTs. SBFT is relatively easy to perform and is tolerable for patients. However, the sensitivity of SBFT is 30–44%. Enteroclysis has a better diagnostic yield, but it requires a highly skilled radiologist and may cause significant discomfort for patients. Despite the advances in CT technology, conventional CT scans still detect only large tumors greater than 10 mm in diameter. In comparison, the diagnostic yield of CE was superior to those of CT and SBFT [30, 31]. The diagnostic impact of CE for SBTs was reported as 52.6% [22]. CE also allows endoscopic imaging of the entire small bowel without discomfort or exposure to radiation. However, CE is contraindicated for patients with suspected or documented intestinal obstruction and presents no opportunity for histological confirmation or therapeutic intervention. Our results demonstrated that CE has a higher diagnostic yield and sensitivity than those of other radiologic modalities.

We demonstrated that the miss rate of CE for SBTs was 16.5%. We also found that missed tumors were most commonly located in the proximal jejunum (55.6%). A meta-analysis including 24 studies and over 500 patients found that the overall miss rate of CE for small bowel mass lesions was 18.6% [32]. Ross et al. reported that CE identified the mass lesion in only 5 (33%) of 15 patients with SBTs [17]. There are several possible explanations for nondetection of SBTs by CE. SBTs are usually single, whereas most patients with angiodysplasia or ulcers have multiple lesions. SBTs are usually located in the proximal small bowel and may be missed because of tumbling or excessive capsule transfer velocity due to excessive bowel peristalsis. Therefore, the detection of SBTs by CE remains a challenge despite CE being a major advance in small bowel evaluation.

Our study is limited by its retrospective design. Furthermore, because our study included only patients with proven SBTs, the specificity of each diagnostic method could not be analyzed. There are limitations to measure accurate sensitivity or miss rate of CE because the patients with negative CE results were more likely not to undergo DBE or surgery.

Taken together, we concluded that CE shows higher diagnostic yield and sensitivity for the diagnosis of SBTs compared with other radiologic tests. However, CE can miss some significant tumors, particularly those located in the proximal jejunum. Further endoscopic investigation by deep enteroscopy should be considered for patients with nonspecific findings on CE but high clinical suspicion for SBT.

## Figures and Tables

**Table 1:** Clinical characteristics of patients with small bowel tumors and characteristics of small bowel tumors.

Clinical characteristics	*N* (%)
M : F	50 : 29
Age (years, mean ± SD)	47.2 ± 20.2
Mean duration of symptoms (days ± SD)	168.9 ± 966.6
Smoking (current)	13 (16.4%)
Alcohol (current)	17 (21.5%)
Comorbidity	
Hypertension	7 (8.9%)
Diabetes mellitus	2 (2.5%)
Liver cirrhosis	1 (1.3%)
Others	14 (17.8%)
Hb (g/dL, mean ± SD)	10.1 ± 2.9
Anemia	45 (57%)
Protein (g/dL, mean ± SD)	6.1 ± 0.9
Albumin (g/dL, mean ± SD)	3.7 ± 0.6
Characteristics of small bowel tumors	
Single tumor	54 (68.3%)
Size of tumor (cm, mean ± SD)	3.5 ± 2.6
Extents	
Localized	68 (86.1%)
Locally advanced	6 (7.6%)
Metastasis	5 (6.3%)

**Table 2:** Comparison of diagnostic yields of CE and other radiologic studies.

	CT	SBFT	CE	DBE
Definitive	38 (55.8%)	18 (46.1%)	45 (83.3%)	61 (93.8%)
Suspicious	20 (29.4%)	9 (23.1%)	2 (3.7%)	
Negative	10 (14.7%)	12 (30.7%)	7 (12.9%)	4 (6.2%)
Total	68	39	54	65

CE: capsule endoscopy; SBFT: small bowel follow-through; CT: computed tomography; DBE: double balloon enteroscopy.

**Table 3:** Comparison of diagnostic yields of capsule endoscopy and other radiologic studies.

	Difference (%)	95% CI (%)	*P* value
CE versus SBFT	36.36	9.36–50.64	0.0075
CE versus CT	33.33	12.75–43.82	0.0015

CE: capsule endoscopy; SBFT: small bowel follow-through; CT: computed tomography.

**Table 4:** Clinical factors affecting diagnostic yields of each modality.

Clinical factors		CT	SBFT	CE
Anemia	Presence	52.5%	48%	75%
	Absence	16.2%	30%	85.7%
	*P* value	0.019	0.333	0.638

Size of tumor	<10 mm	30%	14.2%	77.7%
	>10 mm	37.5%	46.1%	60%
	*P* value	0.900	0.043	0.624

Main symptoms	Bleeding	57.6%	50%	66.6%
	Nonbleeding	42.4%	50%	68.9%
	*P* value	0.021	0.458	0.615

Location	*P* value	0.054	0.546	0.485

CE: capsule endoscopy; SBFT: small bowel follow-through; CT: computed tomography.

**Table 5:** Comparisons of sensitivities of three modalities for small bowel tumors.

	CE			Total	*P* value
	Definitive	Suspicious	Negative		
CT					0.005
Definitive	10	1	1	12	
Suspicious	13	0	5	18	
Negative	1	1	0	2	
Total	24	2	6	32	
SBFT					0.046
Definitive	10	0	3	13	
Suspicious	5	1	1	7	
Negative	9	1	2	12	
Total	24	2	6	32	

CE: capsule endoscopy; SBFT: small bowel follow-through; CT: computed tomography.
